# Effects of Fatty Alcohols with Different Chain Lengths on the Performance of Low pH Biomass-Based Foams for Radioactive Decontamination

**DOI:** 10.3390/molecules27196627

**Published:** 2022-10-06

**Authors:** Hao Zhang, Lili Liang, Hailing Xi, Datong Liu, Zhanguo Li, Xiaoyan Lin

**Affiliations:** 1 Engineering Research Center of Biomass Materials, Ministry of Education, School of Materials Science and Engineering, Southwest University of Science and Technology, Mianyang 621010, China; 2 School of Science, Xichang University, Xichang 615000, China; 3 State Key Laboratory of NBC Protection for Civilian, Beijing 102205, China

**Keywords:** foam decontamination, radioactive pollution, low pH, chain-length matching, biomass-based

## Abstract

Compared with polymers and nanoparticles, fatty alcohols can not only increase the stability of foam, but also maintain better foamability at pH < 2, which is beneficial to reduce waste liquid and increase decontamination efficiency for radioactive surface pollution. However, different fatty alcohols have different hydrophobic chain lengths. The effects of fatty alcohols with different chain lengths on the performance of decontamination foam were studied at pH < 2, to assist in the selection of suitable fatty alcohols as foam stabilizers. Combined with betaine surfactant and phytic acid, biomass-based foams were synthesized using fatty alcohols with different chain lengths. When the hydrophobic tail groups of the fatty alcohol and the surfactant were the same, the foam showed the best performance, including the lowest surface tension, the highest liquid film strength, the greatest sag-resistance and the best stability. However, when the hydrophobic tail groups were different, the space between adjacent surface active molecules was increased by thermal motion of the excess terminal tail segments (a tail-wagging effect), and the adsorption density reduced on the gas-liquid interface, leading to increased surface tension and decreased liquid film strength, sag-resistance and stability. The use of decontamination foam stabilized by fatty alcohols with the same hydrophobic group as the surfactant was found to increase the decontamination rate of radioactive uranium pollution from 64 to over 90% on a vertical surface.

## 1. Introduction

With the reduced availability of fossil energy and increasing global warming, clean, low-carbon and efficient nuclear energy sources are being rapidly developed in many countries [1]. Although the safety of nuclear energy is high today, there is still a risk of nuclear explosions and leakage, as manifest in the Chernobyl nuclear explosion and the Fukushima nuclear accident [2]. Inevitably, many surfaces contaminated by radioactive radionuclides occurs during the operation of a nuclear power plant [3]. In addition, the potential for widespread radioactive contamination resulting from terrorist attack is increasing in cities [2,4]. Therefore, the decontamination of radioactive polluted surfaces is an important part of nuclear security.

Many physical or chemical methods are used for surface decontamination, but environmental remediation in large-scale surface radioactive contamination is extremely challenging because of the shortcomings of these methods. For example, the commonly used high-pressure water method has poor removal effects and also produces a large amount of waste liquid carrying radionuclides [5]. Moreover, if the waste liquid is not collected in time, it may penetrate porous media or flow into water, causing secondary pollution that is more difficult to handle. Although a grinder or blast is commonly used, these may damage the structure of the object and generate radioactive dust, and are not suitable for irregular surfaces [4,5]. Lasers with low decontamination efficiency consume a lot of energy and require special equipment [6]. Ionic washing accelerates the ion exchange of radionuclides, but its effectiveness for removal is limited [7]. Recently, strippable coatings have been increasingly studied [4,5]. However, it takes a long time to dry the coatings, and special procedures are required to strip the coatings after the radionuclides are adsorbed. Furthermore, the decontamination methods mentioned have difficulty dealing with challenging surfaces, including sloped, vertical, ceiling, and irregular surfaces and large cavity interiors.

Foam is a two-phase system composed of gas and liquid. Owing to its special physical and chemical properties, it was used in the decontamination of decommissioned nuclear facilities in France as early as the 1990s [8]. Foam decontamination mainly depends on the foam carrying chemical reagents with excellent solubility for radioactive pollutants to clean the surface [8,9,10]. The gas constitutes a large volume fraction of the foam, resulting in rapid decrease in the amount of liquid after defoaming, and reducing the cost of waste liquid treatment [8,11]. The ability to visualize foam decontamination is high, the surface covered by foam being obviously white [12], which can help avoid repetition and omission when spraying foam, and improve decontamination accuracy. The equipment needed for foam decontamination is widely available and inexpensive. A large amount of foam can be produced using a foam car washer, and the foam can be collected using a vacuum cleaner after decontamination [12]. In addition, after the foaming solution becomes foam, the viscosity increases markedly and the density decreases significantly, which means that foam has excellent sag-resistance and adheres to surfaces for effective decontamination, including sloped, vertical, ceiling and irregular surfaces and large cavity interiors [8,11,12]. This is an advantage that is unmatched by any other decontamination method.

However, foam is a thermodynamically unstable system that decays continuously after formation, and eventually the liquid and gas separate completely [13]. In order to prevent foam from decaying, nanoparticles, proteins and polymers can be added to improve foam stability. Although these foam stabilizers can significantly increase the stability of foam, they can markedly decrease foamability [14,15,16], which increases the volume of waste liquid and weakens the sag-resistance. In addition, common surface radioactive pollutants mainly exist in the form of metal salts and metal oxides, which are readily dissolved in the foam at pH < 2 [9]. However, the low pH may affect the foamability of surfactants and the stability of the foam stabilizers. Therefore, the features and performance of the surfactants and stabilizers at low pH have important effects on the performance of decontamination foam. Due to the presence of both positive and negative charges in the hydrophilic head group, the mildly toxic and easily biodegradable betaine surfactant has good foamability in strong acid or strong alkaline environments [17], making it an ideal foaming agent at low pH. Moreover, fatty alcohols synthesized and degraded by microorganisms [18] can greatly increase foam stability without reducing foamability at suitable concentrations [19]. Moreover, the non-ionized hydroxyl group is not easily affected by ions in solution and can act as a stabilizer at low pH. However, little research has been conducted on the effect of fatty alcohols with different chain lengths on low pH foam performance. Phytic acid extracted from plants [20] has a strong chelating effect on radioactive metal ions at low pH [21,22], which can improve the dissolution efficiency of radioactive contaminants on a surface. It is very suitable for use as a pH conditioning agent. In this study, using betaine surfactant as a foaming agent and phytic acid as a pH conditioning agent, the effect of fatty alcohols with different chain lengths on the performance of decontamination foam was investigated.

## 2. Results and Discussion

### 2.1. Effect on Drainage Stability and Foamability of Fatty Alcohols with Different Chain Lengths

**Figure 1:**
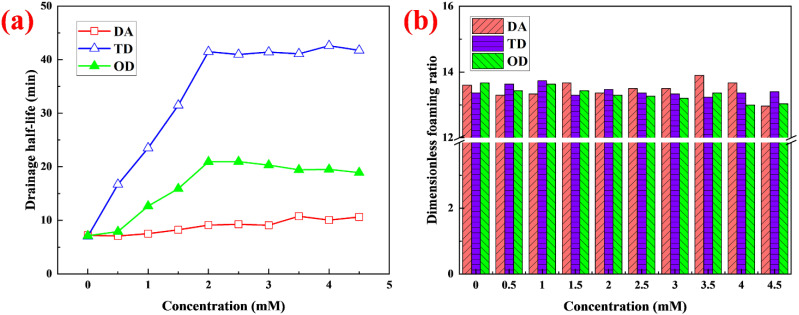
(**a**) T_h_ and (**b**) R_f_ at different concentrations.

Three fatty alcohols with different chain lengths, DA, TD, and OD, were used as the foam stabilizers. The drainage stability and foamability of the foam were measured at different concentrations at pH 1.8 (Figure 1). As the concentration of DA increased, the T_h_ value of the foam improved slightly (from 7 min to 10 min) (Figure 1a). As the concentration of OD increased, the T_h_ value tended to rapidly increase at first and then to change little. When the concentration reached 2 mM, T_h_ was 21 min, which was three times that of the foam without stabilizer. As the concentration of TD increased, T_h_ also tended to increase rapidly at first and then change little, but the increase in T_h_ of the foam stabilized with TD was greater than that with OD. The T_h_ of the foam stabilized by TD was 41 min at 2 mM, which was about six times that of foam without stabilizer. Although the three fatty alcohols with different chain lengths were able to improve the stability of the foam, their effects were obviously different. The foam stability of TD was the best, followed by OD, with DA showing the least foam stability. The chain lengths and concentrations of fatty alcohols had little effect on the dimensionless foaming ratio (foamability) and R_f_ was mostly between 13 and 14 (Figure 1b). However, the foamability of the foaming solution stabilized by fatty alcohols was significantly different from that of foams stabilized by nanoparticles [16], proteins [15] and polymers [14]. Although nanoparticles, proteins and polymers can increase the stability of the foams, the foamability of the foaming solution greatly reduces with increasing concentration. For decontamination foam, a significant reduction in foamability is unfavorable because of the following effects: (1) If R_f_ is reduced by half, the volume of the waste liquid will double after the same volume of foam is defoamed, which will signally increase the treatment cost of radioactive waste liquid [8,10]; (2) If R_f_ is reduced by half, the gravity of foam having the same volume will double, which adversely affects adherence on vertical surfaces. The sag-resistance may be reduced, and foam may quickly slide off vertical surfaces, resulting in decreased reaction time and a reduced decontamination rate [12]. Therefore, suitable chain lengths and appropriate concentrations of fatty alcohols can not only greatly improve stability, resulting in prolonging the interaction time of foam with the contaminated surface, but also not weaken foamability, ensuring low waste liquid volume and good sag-resistance (discussed further in Section 2.8 and Section 2.9).

### 2.2. Effect on Surface Properties of the Foaming Solution by Fatty Alcohols with Different Chain Lengths

**Figure 2:**
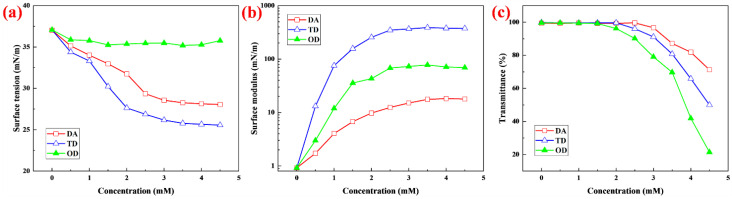
(**a**) Surface tension, (**b**) surface modulus, and (**c**) transmittance at different concentrations.

To study the effects of fatty alcohols with different chain lengths, such as DA, TD, and OD, on the surface properties of the foaming solution, the surface tension and surface modulus were measured for different concentrations of the fatty alcohols. The surface tension tended to first decrease and then change little as the concentration of the fatty alcohols increased, but the decreasing amplitude for the different fatty alcohols was very diverse (Figure 2a). For OD, the surface tension of the foaming solution decreased from 37.1 mN/m to 35.3 mN/m as the concentration of OD increased from 0 to 1.5 mM, and then the surface tension changed little. For TD, the surface tension of the foaming solution decreased greatly as the concentration of TD increased from 0 to 3.5 mM. When the surface tension decreased to 25.6 mN/m, there was little change with increase in TD concentration. Among the three fatty alcohols, TD caused the greatest decrease in surface tension. For DA, the surface tension of the foaming solution decreased significantly as the concentration of DA increased from 0 to 3.5 mM, but the decreasing amplitude was less than that of TD. For solutions of the same system, the lower the surface tension, the greater the adsorption density of surface active molecules [23]. According to the surface tension, TD increased the adsorption density the most, DA next, and OD increased adsorption density the least.

The stability of a bubble liquid film is determined more by the surface modulus than the surface tension. Surface modulus is a parameter reflecting the strength of liquid film. The larger the surface modulus, the more stable the liquid film [24]. There is no necessary relationship between surface tension and surface modulus, and a solution with low surface tension may not have a high surface modulus [25]. There are three alternatives: (1) solutions with high surface tension and low surface modulus; (2) solutions with low surface tension and low surface modulus; and (3) solutions with low surface tension and high surface modulus [25]. The surface modulus of the foaming solution was measured at different concentrations of DA, TD, and OD (Figure 2b). With increase in concentration, the surface modulus for all three fatty alcohols tended to rapidly increase at first and then to change little, but the increase in amplitude of the different fatty alcohols was very different. For DA, when the surface modulus reached 17.6 mN/m, there was little change with increase in the concentration of DA. For TD, when the surface modulus reached 367.8 mN/m, there was little change with increase in the concentration of TD. Compared with the solution without fatty alcohols, the surface modulus increased 399-fold. Among the three fatty alcohols, TD caused the greatest increase in surface modulus. For OD, when the surface modulus reached 72.8 mN/m, there was little change with increase in OD concentration. The order of surface modulus was the same as that of the drainage stability (Figure 1a) and the bubble stability (Figure in Section 2.6) (discussed further in Section 2.6).

### 2.3. Solubility of Fatty Alcohols with Different Chain Lengths

**Figure 3:**
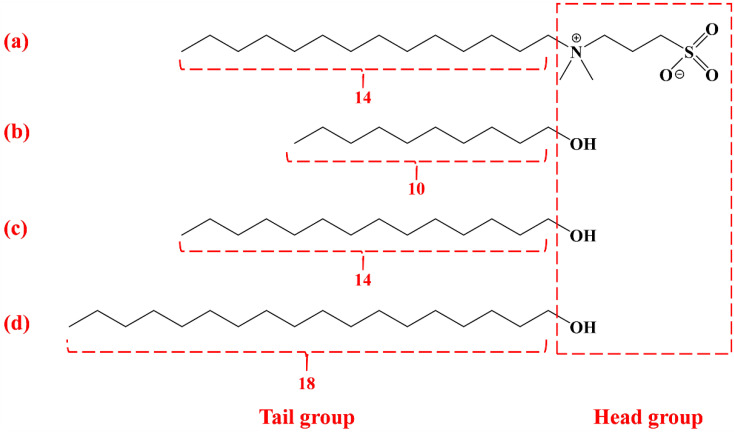
Structural formula of (**a**) DMAPS (**b**) DA (**c**) TD and (**d**) OD.

DA, TD, and OD have structures similar to the tail and head groups of DMAPS surfactants (Figure 3). However, these fatty alcohols are hardly dissolved in water due to the weak polarity of the head group [26], which limits their application. An effective method to improve the solubility of long-chain fatty alcohols is to form mixed micelles with surfactant which are then dissolved in the surfactant solution [26]. This can significantly affect the surface and bulk phase properties of the solution [27,28], such as the surface tension, surface modulus and solution viscosity (Section 2.2 and Section 2.4). DA, TD, and OD were separately dissolved in DMAPS solution containing PA at pH 1.8. The transmittance of the DMAPS solution was measured (Figure 2c). With increase in concentration, the transmittance of the solution tended first to remain constant (close to 100%) and then to decrease. However, when the transmittance of the solution began to decline, the corresponding concentration of DA, TD, and OD was different, indicating that the solubility of fatty alcohols with different chain lengths was different. The solubilities of DA, TD, and OD were 3 mM, 2.5 mm and 2 mM, respectively. Solubility decreased with increase in chain length of the fatty alcohols, showing normal chain length dependence [26]. The concentration of the betaine surfactant (DMAPS) was about 27.5 mM, which was much greater than the solubility of the fatty alcohols, so fatty alcohols are cosurfactants [27]. After the dissolution of the fatty alcohols reached saturation, change in concentration of the fatty alcohols had little effect on the surface and bulk phase properties of the DMAPS solution [26,27,28]. Therefore, the saturated fatty alcohol solutions were used for the following experiments.

### 2.4. Effect of Fatty Alcohols with Different Chain Lengths on the Solution and Foam Viscosity

**Figure 4:**
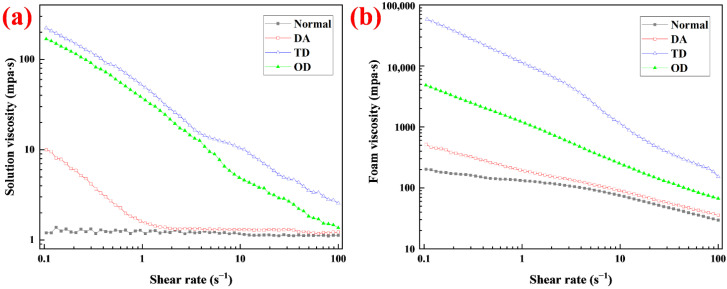
Apparent viscosity of (**a**) solutions and (**b**) foams at different shear rate. The label “Normal” represents the solution and foam without fatty alcohols.

Fatty alcohols dissolved in surfactant solutions not only affect surface properties (Section 2.2) but may also affect the bulk phase properties of the solution. The apparent viscosity of surfactant solutions with different fatty alcohols was measured for different shear rates (Figure 4a). As the shear rate increased from 0.1 s^−1^ to 100 s^−1^, the apparent viscosity of the different solutions exhibited different characteristics. For the normal solution (foaming solution without fatty alcohols), the apparent viscosity was always low and did not change greatly with increasing shear rate, exhibiting Newtonian fluid characteristics. For DA, the apparent viscosity decreased rapidly when the shear rate increased from 0.1 s^−1^ to 1 s^−1^, and then the apparent viscosity changed little. For both TD and OD, the apparent viscosity decreased gradually with increasing shear rate, showing obvious shear-thinning behavior. Shear-thinning is a rheological characteristic of polymer solutions occurring when the network structure formed by the polymer is destroyed with increasing shear rate [29]. Nevertheless, all components in these solutions were small molecules, which should show shear characteristic of Newtonian fluids; their apparent viscosity should not change with increasing shear rate. However, the apparent viscosity of the solutions containing TD and OD exhibited shear-thinning of the polymer solutions, indicating that small molecules self-assembled to worm-like micelles. The worm-like micelles intersect and entangle each other, forming a network structure similar to a polymer solution, which increases the viscosity of the solution. When the shear rate increases, the network structure formed by the worm-like micelles is destroyed and the apparent viscosity decreases [29,30]. Many studies have shown that fatty alcohols can form worm-like micelles with surfactants and increase system viscosity [30,31,32]. In similar systems, the longer the wormlike micelles, the greater the apparent viscosity at the same shear rate [33]. For DA, shear-thinning occurred at shear rates of 0.1 s^−1^ to 1 s^−1^. However, the apparent viscosity of the solution was much lower than that of solutions with TD and OD at the same shear rate. When the shear rate was greater than 1 s^−1^, the apparent viscosity of the solution with DA was almost unchanged, showing the characteristics of Newtonian fluids. This indicates that the network structure formed by the worm-like micelles was completely destroyed in the solution with DA. According to the apparent viscosity of the solution, the length of the worm-like micelles formed by the fatty alcohols and the surfactant was in the order: TD > OD > DA.

**Figure 5:**
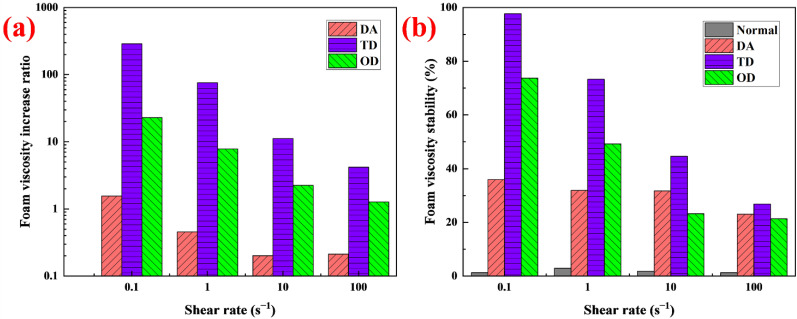
(**a**) I_fv_ and (**b**) S_fv_ at different shear rate.

After a surfactant solution turns into foam, a single-phase system composed of liquid transforms into a two-phase system composed of gas and liquid. The rheological properties change significantly, the apparent viscosity increases dramatically, and shear thinning occurs [34]. The apparent viscosity of the foams was measured at different shear rates (Figure 4b). The apparent viscosity of all foams decreased with increasing shear rate. Compared with the corresponding solutions, the apparent viscosity of the foam increased significantly at the same shear rate, especially the foam stabilized by TD, whose apparent viscosity increased by more than two orders of magnitude. At the same shear rate, the order of foam apparent viscosity was: TD > OD > DA > normal. This was of the same order of magnitude as the apparent viscosity of the corresponding solution, since high solution viscosity tends to correspond to high foam viscosity [35]. At the same shear rate, the foam viscosity increase ratio $I_{fv}=\mu_{i}/\mu_{0}-1$ was used to indicate the increased multiple of foam viscosity after adding the stabilizer. where µ_i_ is the viscosity of foam containing stabilizer, mpa·s; and µ_0_ is the viscosity of foam without stabilizer, mpa·s. At the same shear rate, I_fv_ of TD was always the largest among the three fatty alcohols (Figure 5a), that is, TD thickened the foam the most. The thickening effect of the fatty alcohols on the foam decreased rapidly with increasing shear rate. For decontamination foam, the foam is formed at high shear rates, and, if the apparent viscosity of the foam is low at this time, it facilitates the formation and transportation of foam in the pipeline [8]. After the foam was sprayed on the surface, the flow of the foam on the vertical surface tended to slow down due to the absence of an external force, and the corresponding shear rate decreased. As the apparent viscosity increased at a low shear rate, this helped to prevent the foam from flowing downwards, leading to prolonging of the reaction time on the vertical surface and improving the decontamination rate [8,10,12]. In this study, TD had no obvious thickening effect on the foam at high shear rates, which was favorable for the formation and transportation of foam. However, TD had a significant thickening effect on the foam at low shear rates, which was beneficial for improving the sag-resistance and prolonging the reaction time. The effects of the application of TD thickening foam at different shear rates is discussed further in Section 2.8.

### 2.5. Effect of Fatty Alcohols with Different Chain Lengths on Foam Viscosity Stability

**Figure 6:**
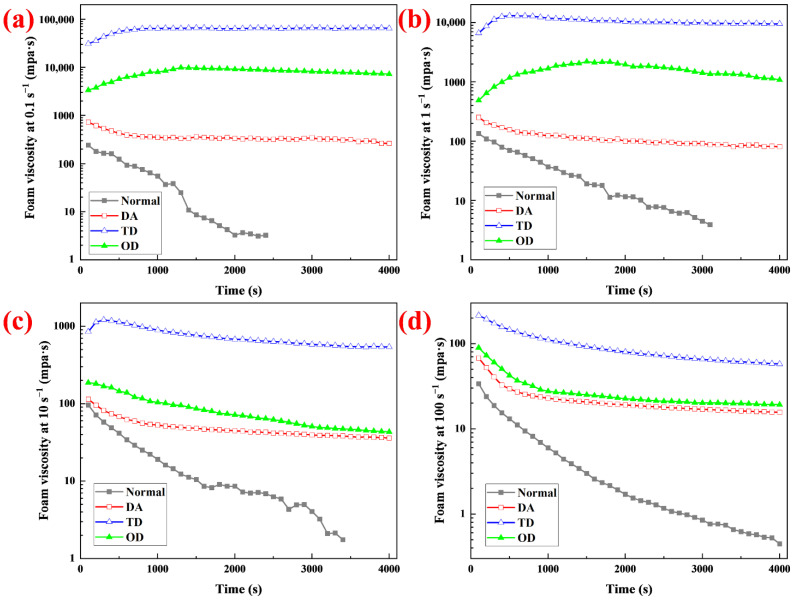
Apparent viscosity of foams as a function of time at different shear rates (**a**) 0.1 s^−1^, (**b**) 1 s^−1^, (**c**) 10 s^−1^ and (**d**) 100 s^−1^.

Foam is a thermodynamically unstable system, which decays continuously after forming [13]; its rheological properties change with increasing time [34]. If the foam is sprayed on a vertical surface, the decay may cause the viscosity to decrease so much that the foam slides off, resulting in shortening of the decontamination time. In Section 2.4, it was demonstrated experimentally that fatty alcohols can increase the foam viscosity; in particular, TD enhanced the foam viscosity by more than two orders of magnitude at low shear rates. However, whether the viscosity stability of the foam is also improved needs further study. The viscosity changes of the foams with time at different shear rates were measured within 4000 s (Figure 6). The viscosity of normal foam decreased with increasing time at different shear rates. At 0.1 s^−1^, 1 s^−1^ and 10 s^−1^, the viscosity dropped to almost untestable levels before the time reached 4000 s but showed a breakpoint. After the breakpoint, the viscosity fluctuated greatly, and even had a negative value, so these viscosities were discarded. The viscosity of the foam containing DA decreased with increasing time at different shear rates. At 0.1 s^−1^ and 1 s^−1^, the viscosity of the foam containing OD first increased and then decreased with increasing time. The high viscosity foam with OD did not spread fully on the test bench at the beginning. With continuous rotation of the turntable, the foam gradually spread out on the test bench, the contact area between the foam and the turntable increased, and the measured viscosity increased. The decrease in viscosity was due to the continuous decay of the foam during the test. Compared with the viscosity of the foam at 0.1 s^−1^ and 1 s^−1^, the viscosity at 10 s^−1^ and 100 s^−1^ always decreased with increasing time. At higher shear rates, the turntable rotated faster and the foam with OD quickly spread out on the test bench. The contact area between the foam and the turntable reached the maximum in a short time, and the first test point was maximum viscosity. For TD, at 0.1 s^−1^, the viscosity of the foam first increased and then hardly changed with increasing time. At 1 s^−1^ and 10 s^−1^, the viscosity of the TD foam first increased and then decreased. At 100 s^−1^, the viscosity of the TD foam always decreased. The foam viscosity stability was evaluated by the formula $S_{\mathrm{fv}}=\left(\mu_{\mathrm{end}}/\mu_{\max}\right)\times 100\%$. where µ_end_ refers to the viscosity at the end of the test, mpa·s; µ_max_ refers to the maximum viscosity, mpa·s. For normal foam, the viscosity at breakpoint was used as the µ_end_. Figure 5b shows the viscosity stability of different foams within 4000 s. Compared with the foam without fatty alcohol, all the viscosity stabilities of the foam with fatty alcohol were significantly raised, indicating that fatty alcohols can improve foam viscosity stability. Among the fatty alcohols with different chain lengths, TD enhanced foam viscosity stability the most, reaching 97.7% at 0.1s^−1^. The application of the viscosity stability associated with TD foam is discussed further in Section 2.8.

### 2.6. Effects of Fatty Alcohols with Different Chain Lengths on Foam Microstructure

**Figure 7:**
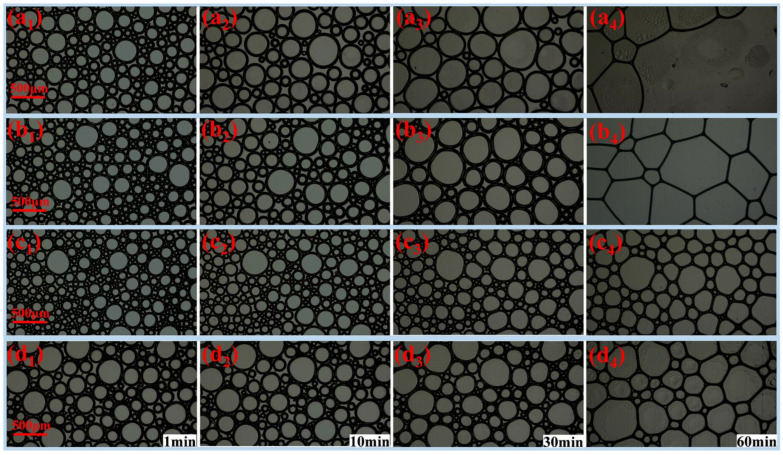
Foam microstructure at different time: (**a**) normal foam, (**b**) DA foam, (**c**) TD foam and (**d**) OD foam. The subscripts 1, 2, 3 and 4 refer to 1 min, 10 min, 30 min and 60 min, respectively.

**Figure 8:**
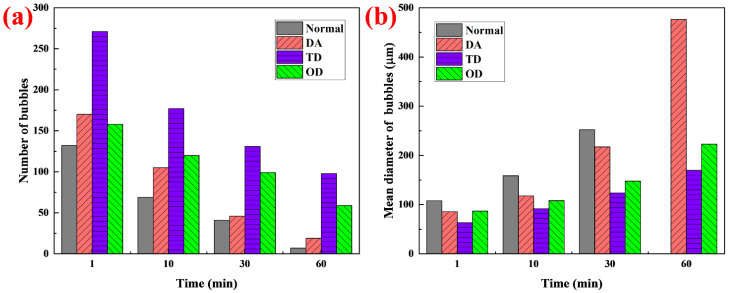
(**a**) number and (**b**) mean diameter of bubbles at different times.

Foam has a large total surface area after formation and, therefore, has a high surface energy. To reduce the total surface energy, the total surface area is reduced spontaneously through coarsening, coalescence and drainage to decrease the total number of bubbles and increase the mean size of the bubbles [24,36]. To investigate whether fatty alcohols can slow down this decay process, the microstructure changes of the bubbles were analyzed within 60 min (Figure 7). With increasing time, the number of bubbles decreased and the mean diameter increased in all foams, showing obvious decay characteristic [24,36]. However, different foams had different decay rates. The number and mean diameter of the bubbles were calculated using graphic analysis software; the results are shown in Figure 8. Compared with normal foam without fatty alcohol, fatty alcohol can increase the number of bubbles (Figure 8a); TD increased the number of bubbles the most, which indicates that TD can effectively inhibit the decay of foam at the microscopic level. TD can significantly reduce the drainage (Section 2.1), markedly increase the surface adsorption density (Section 2.2), and increase bubble liquid film strength (Section 2.2) and solution viscosity (Section 2.4), thus, effectively resisting gas diffusion from small bubbles to large bubbles, resulting in considerable improvement in foam stability. Compared with normal foam without fatty alcohol, fatty alcohols can reduce the mean diameter of the bubbles (Figure 8b); in particular, the foam with TD had the smallest mean diameter of bubbles. The bubbles without fatty alcohols ruptured severely at 60 min (Figure 7a_4_), resulting in the bubbles not covering the entire observation area. Therefore, the mean bubble diameter was not calculated at 60 min. The increased rate of bubble mean diameter was calculated within 60 min using the formula $R_{md}=\left({\bar{D}}_{2}-{\bar{D}}_{1}\right)/\left(t_{2}-t_{1}\right)$, where, ${\bar{D}}_{1}$ and ${\bar{D}}_{2}$ refer to the mean bubble diameter at t_1_ and t_2_, μm. The R_md_ values of the foam stabilized by DA, TD and OD were 6.51, 1.77 and 2.27 μm·min^−1^, respectively. TD foam had the smallest R_md_, which indicates that TD can effectively prevent the bubbles from becoming larger, thus improving the stability of the foam at the microscopic level.

### 2.7. Foam Stabilization Mechanism of Fatty Alcohols with Different Chain Lengths

**Figure 9:**
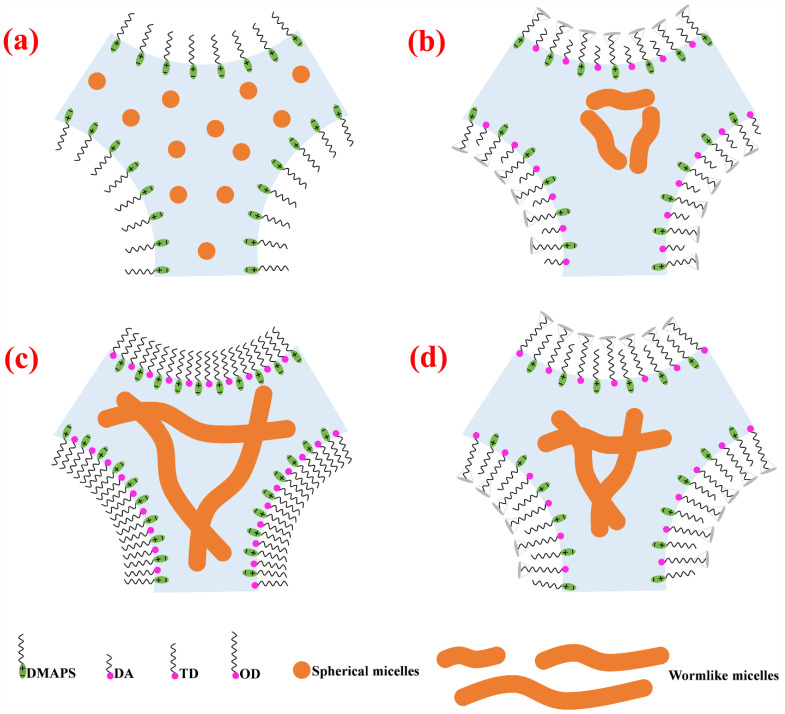
Schematic diagram of the foam stabilized by fatty alcohols: (**a**) the absence of fatty alcohol; (**b**) DA; (**c**) TD and (**d**) OD.

Based on the outcomes of the experiments described in Section 2.1, Section 2.2, Section 2.3, Section 2.4, Section 2.5 and Section 2.6, analysis indicated that fatty alcohols with different chain lengths affected the surface and bulk phase properties of foam. In the foam without fatty alcohol, only the surfactant was adsorbed on the gas-liquid interface, and the surfactant only formed spherical micelles in the liquid film (Figure 9a). Fatty alcohols have hydrophilic head groups and hydrophobic tail groups (Figure 3), which is similar to the structure of surfactants. After adding fatty alcohol to the surfactant solution, the fatty alcohol is adsorbed together with the surfactant on the gas-liquid interface [37]. Since the head group of the fatty alcohol is a hydroxyl group, it is difficult to ionize in solution. When the fatty alcohol is between the chains of surfactant molecules, to a certain extent its uncharged head group shields the electrostatic repulsion between the head groups of the surfactant. As a result, the space between adjacent surface active molecules is shortened and they become more closely aligned on the gas-liquid interface, thus increasing the adsorption density (Figure 9c). However, if the hydrophobic tail length of the surfactant and the fatty alcohol is different, the space between adjacent molecules will increase by thermal motion of the excess terminal tail segments (a tail-wagging effect) [23,37]. The electrostatic shielding effect produced by the head group of the fatty alcohol is, to a certain extent, counteracted, so that the molecules are not as closely arranged as the molecules with the same tail group on the gas-liquid interface (Figure 9b,d). This is also the reason why the TD solution had a lower surface tension, while the DA and OD solutions showed higher surface tension. Fatty alcohols affect not only the surface properties but also the bulk phase properties. It has been reported that cosurfactants (such as fatty alcohols) can form worm-like micelles with surfactants [30,31,32]. The micelle morphology formed by a surfactant is related to the packing parameter p=V/al [38], where V is the volume of the hydrophobic part of the surfactant, a is the effective surface occupied by the surfactant head group, and l is the length of the hydrophobic tail. In general, *p* < 1/3 in pure surfactant solution, and the micelle is spherical [39]. After the addition of fatty alcohol, the electrostatic repulsion between the surfactant head groups is to a certain extent shielded, the distance between the surfactants is shortened, and the effective area of the head group is reduced, which increases the stacking parameter. When 1/3 < *p* < 1/2, worm-like micelles may form [38,39], thus changing the viscosity of the solution. As indicated in Section 2.4, after the addition of fatty alcohol, the occurrence of shear-thinning behavior also indicates that wormlike micelles may form. The lengths of the worm-like micelles formed by the fatty alcohol and surfactant were in the order: TD > OD > DA.

### 2.8. Foam Sag-Resistance Study

**Figure 10:**
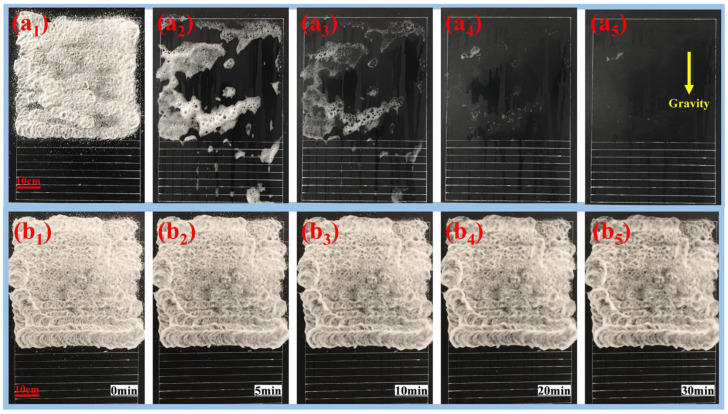
The sag-resistance of (**a**) normal foam and (**b**) foam stabilized by TD on vertical surface. The subscripts 1, 2, 3, 4 and 5 refer to 1 min, 5 min, 10 min, 20 min and 30 min, respectively.

To test the interaction time between the foam and the vertical surface, sag-resistance was measured (Figure 10). Normal foam without TD and foam stabilized by TD were each sprayed on an area of 50 cm × 50 cm and the flow of the foams on the vertical surface was tested for 30 min. The space between the adjacent lines was 3 cm. In the same duration, the more stripes the foam flowed through, the worse the sag-resistance. For the foam without fatty alcohol, the foam flowed through almost all the stripes; more than half of the area was not covered by the foam within 5 min as the foam slid down. The sag-resistance of the foam without fatty alcohol was poor—the effective interaction time between the foam and the vertical surface was less than 5 min. However, the foam stabilized by TD hardly flowed within 30 min and always adhered to the sprayed area within 30 min, indicating that the foam stabilized by TD had good sag-resistance, which significantly extended the decontamination time on the vertical surface. The excellent sag-resistance of the foam stabilized by TD was determined by two factors: (1) TD can significantly increase foam stability without reducing foamability; (2) TD can increase the foam viscosity by more than two orders of magnitude, which greatly enhances resistance to flow.

### 2.9. Foam Decontamination Analysis

**Figure 11:**
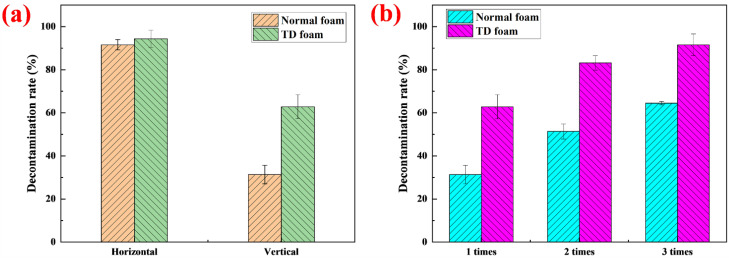
(**a**) Decontamination rate on horizontal and vertical surfaces. (**b**) Decontamination rate of normal foam and TD foam on vertical surface.

To test the removal ability of foam detergents for simulated uranium contamination on the surface of stainless-steel plates at different angles, the surface radioactive pollution on the horizontal and vertical plates was decontaminated separately; the results are shown in Figure 11. For the horizontal surface, the decontamination rates of normal foam and the foam stabilized by TD were both above 90%, and there were no significant differences in the decontamination rates. During the whole decontamination process, the foam and drainage liquid were always in contact with the radioactive pollutants on the horizontal surface, which increased the dissolution time. Hence, the decontamination rate of the horizontal surface was high. However, for the vertical surface, the decontamination rates were diverse; normal foam without TD comprised only about half of the foam stabilized by TD. Because the sag-resistance of normal foam without TD was poor, the foam flowed down quickly after being sprayed on the vertical surface. This severely shortened the interaction time between the foam and the contaminant on the vertical surface, resulting in a low decontamination rate. After the foam stabilized by TD was sprayed on the vertical surface, it hardly flowed down because of the excellent sag-resistance of the foam, leading to significant increase in the contact time and the decontamination rate. However, compared with the horizontal surface, the single decontamination rate of TD foam was significantly lower on the vertical surface, being only about 63%. Although TD foam hardly flowed on the vertical surface, the TD foams slowly released liquids that flowed downwards, resulting in less dissolved contaminants and lower decontamination rates. To improve the decontamination rate, the vertical surface was decontaminated several times [10]. After three decontamination periods on the vertical surface, the decontamination rate of TD foam reached more than 90%, while that of the normal foam was only 64%.

## 3. Materials and Methods

### 3.1. Materials

Phytic acid solution (PA, 50% in H_2_O), n-Decanol (DA, 98%), n-Tetradecanol (TD, 98%), n-Octadecanol (OD, 98%), and 3-(N,N-Dimethylmyristylammonio)propanesulfonate (DMAPS, 98%) were purchased from Aladdin Biochemical Technology Co., Ltd. (Shanghai, China). Radioactive contaminants: uranyl nitrate was supplied by Chushengwei Chemical Co., Ltd. (Hubei, China). All reagents were used directly without further purification.

### 3.2. Methods

#### 3.2.1. Preparation of Decontamination Foaming Solutions

DMAPS was dissolved in deionized water to a concentration of 30 mM. In the DMAPS solutions, PA was added to bring pH down to 1.8. Then, DA, TD and OD, respectively, were added to the solutions, to achieve concentrations of these solutions between 0–4.5 mM. All the fatty alcohol solutions, with different concentrations, were stirred at a speed of 500 rmp for 2 h at 65 °C (above DA, TD and OD melting points). Then, the solutions were placed in an incubator for 2 h at 65 °C. After 12 h in the incubator at 20 °C, the next tests were carried out.

#### 3.2.2. Foamability and Drainage Stability Measurement

A 50 mL solution was fully foamed with foam agitator (EW-071, Zheguang Precision Co., Ltd., Shanghai, China) at 12,000 rpm for 1 min. The foam was quickly poured into the measuring cylinder and the volume (V_f_) was recorded. When the foam precipitated 25 mL liquid, the duration was recorded as the half-life (T_h_), which was used to evaluate the drainage stability of the foam [16]. In order to address the effect of different volumes of the foaming solution, the dimensionless foaming ratio $R_{f}=V_{f}/V_{l}$ was used to evaluate the foamability of the foaming solution, where V_f_ refers to the initial foam volume, ml, and V_l_ refers to the foaming solution volume, ml. For each foaming solution, the average values of three independent measurements were used to obtain T_h_ and R_f_. The ambient temperature was controlled at 20 ± 3 °C.

#### 3.2.3. Surface Properties Measurement

The surface tension was measured using a multipurpose tensiometer (SIGMA 700, KVS Instruments Ltd., Helsinki, Finland) using the Du Noüy ring method. The average value of three independent measurements was used to represent the surface tension of the solution at 20 ± 1 °C.

The surface modulus was measured using an automatic interfacial tensiometer (DSA100HP, Krüss, Hamburg, Germany) using the oscillating drop method. The temperature was 20 ± 1 °C, and the oscillation frequency was 0.2 Hz. The average value of three independent measurements was used to obtain the surface modulus of the solution.

#### 3.2.4. Transmittance Measurement

The transmittance of the solution was measured by Multiskan spectrum (1500, Thermo Fisher Scientific Oy, Vantaa, Finland) at a wavelength of 500 nm at 20 ± 1 °C. If there was insoluble matter in the solution, the conical flask was shaken to disperse the insoluble matter before measuring the transmittance [26]. The average value of three independent measurements was used to determine the transmittance of the solution.

#### 3.2.5. Solution and Foam Viscosity Measurement

A 1ml solution was evenly spread on the test bench to avoid bubbles. Solution viscosity was measured using a rotary rheometer (HAAK MARSII, Thermo Fisher Scientific Ltd., Bremen, Germany) with increasing shear rate from 0.1 s^−1^ to 100 s^−1^ at 20 ± 0.5 °C.

In order to eliminate the influence of water content on viscosity, the initial water content of all foams was controlled at about 10% [27,28]. The specific method was as follows: After 10 mL foaming liquid was poured into a 100mL beaker, the solution was foamed using a foam agitator at 12,000 rpm. When the foam volume reached 100 mL, stirring of the solution was stopped immediately. A quantity of 1 mL foam was quickly dropped onto the test bench. The foam viscosity was measured by a rotary rheometer with increasing shear rate from 0.1 s^−1^ to 100 s^−1^ at 20 ± 0.5 °C.

In order to study the viscosity stability of foam, foam with 10% water content was produced using the above method. The viscosity of the foam was measured using a rotary rheometer as a function of time for 4000 s at the fixed shear rate.

#### 3.2.6. Foam Microstructure Analysis

In order to eliminate the effect of water content on the foam microstructure, all foams were required to have same initial water content. After the foam with 10% water content was produced by the method described in Section 3.2.5, a drop of foam was quickly dropped on the glass slide and then pressed by a coverslip. The foam microstructure was observed within 60 min by microscope (DM2500, Lycra Ltd., Kriftel, Germany). Then the number and mean diameter of bubbles were determined using graphical analysis software.

#### 3.2.7. Sag-Resistance Measurement

A test area of 50 cm × 50 cm was drawn on the experiment plate, and then lines with 3 cm intervals were drawn (Figure 10). For the same duration, the more stripes the foam flowed through, the worse sag-resistance was assessed to be. An output pressure of 0.7 Mpa was generated by an air compressor (V-0.17, Lida Machinery Co., Ltd., Quanzhou, China). Foam was sprayed on an area of 50 cm × 50 cm using a foam gun (SG-GC024, Xinge Co., Ltd., Shanghai, China). The flow of the foam on the vertical surface was recorded within 30 min using a video camera.

#### 3.2.8. Foam Decontamination Experiment

The background value A_0_ of the experimental plate surface before pollution was measured using a surface radioactive contamination measuring instrument (FJ-2207, Xi’an Nuclear Instrument Ltd., Xian, China). According to the method described in [14], a radioactive solution (uranyl nitrate solution) was spread evenly on the experimental plate. After drying at 35 °C for 12 h, the surface contamination radioactivity value A_1_ was measured. The foam detergent was sprayed onto the contamination surface using the foam gun, and the foam was removed after 30 min. It was then dried at 35 °C for 12 h and the radioactivity value A_2_ after foam decontamination was measured. The decontamination rate (DR) was calculated according to Equation (1) [12].

(1) $$\mathrm{DR}=\frac{\left(A_{1}-A_{0}\right)-\left(A_{2}-A_{0}\right)}{A_{1}-A_{0}}\times 100\%=\frac{A_{1}-A_{2}}{A_{1}-A_{0}}\times 100\%$$

## 4. Conclusions

Decontamination foam with good stability and strong sag-resistance was synthesized using a compound of betaine surfactant, fatty alcohol and biomass acid. It was determined experimentally that fatty alcohols with different chain lengths affected the surface and bulk phase properties of foam. Due to the chain-length compatibility of TD fatty alcohol with the betaine surfactant [37], TD was able to increase the adsorption density on the gas-liquid interface, and increased the length of the worm-like micelles in the bulk phase. In addition, TD increased the foam viscosity by more than two orders of magnitude, resulting in significantly enhanced sag-resistance of the foam. After the foam stabilized by TD was sprayed on the vertical surface, it hardly flowed within 30 min, which greatly prolonged the interaction time of the foam with the surface contaminated by uranium. Compared with normal foam without TD, the foam stabilized by TD greatly increased the decontamination rate of radioactive pollution on a vertical surface. The decontamination rate of TD foam reached more than 90%, while that of normal foam was only 64% after three periods of decontamination on the vertical surface.

## Data Availability

Data is contained within the article.

## Sample Availability

Samples of the compounds are not available from the authors.
